# Interictal dysphoric disorder in drug-resistant epilepsy: clinical features, cognitive function and quality of life

**DOI:** 10.1192/j.eurpsy.2026.11035

**Published:** 2026-07-31

**Authors:** R. Sánchez-González, E. Monteagudo-Gimeno, L. Pintor-Pérez

**Affiliations:** 1 Department of Psychiatry., Institut de Salut Mental, Centre Emili Mira. Hospital del Mar., Barcelona; 2 Department of Psychiatry, Benito Menni Mental Health Care Complex., Sant Boi de Llobregat; 3 Consultation-Liaison Service. Department of Psychiatry., Institut de Neurociències. Hospital Clínic i Provincial de Barcelona. Institut d’Investigacions Biomèdiques August Pi i Sunyer (IDIBAPS) – Universitat de Barcelona., Barcelona, Spain

## Abstract

**Introduction:**

The term Interictal Dysphoric Disorder (IDD) refers to a pleomorphic affective disorder observed in patients with epilepsy. The prevalence of IDD is believed to be substantial, particularly among individuals with drug-resistant epilepsy (DRE). Nevertheless, the existing literature offers limited evidence regarding the associations between IDD and clinical features, psychiatric disorders (PD), quality of life (QoL) or cognitive function in epilepsy patients.

**Objectives:**

The aim of this study is to investigate the potential relationship between IDD and PD, cognitive function, and quality of life in a cohort of patients with DRE.

**Methods:**

This retrospective study included 281 patients diagnosed with drug-resistant epilepsy (DRE) from the Epilepsy Unit at Hospital Clinic, spanning the years 2008 to 2017. Axis-I psychiatric disorders were assessed using the Structured Clinical Interview for DSM-IV (SCID-IV). Cognitive function was evaluated through a comprehensive battery of tests, including the WAIS III, WMS III, Trail Making Tests A and B, Rey Auditory-Verbal Learning Test, and the Boston Naming Test. Quality of life (QoL) was measured using the Spanish version of the Quality of Life in Epilepsy Inventory-31 (QOLIE-31). Group differences were analyzed using Student’s t-test for continuous variables and the χ^2^ test for categorical variables.

**Results:**

Out of the 281 patients, 97 (34.3%) were diagnosed with Interictal Dysphoric Disorder (IDD), while 184 (65.7%) did not meet criteria for IDD. Among the IDD group, the mean age was 39.2 ± 12.26 years, with a majority being female (59.1%). Of the 97 patients diagnosed with IDD, 70 were also diagnosed with psychiatric disorders (PD) according to the SCID-IV assessment. A significant association was observed between IDD and mood and anxiety disorders (p < 0.05). No significant relationships were found between IDD and other psychiatric disorders. Regarding cognitive function, IDD was significantly associated with lower scores on the vocabulary subtest of the WAIS III (p < 0.05), while no significant associations were identified with other neuropsychological measures. Additionally, the presence of IDD was significantly correlated with lower scores across all domains of the QOLIE-31, indicating a reduced quality of life (p < 0.05).

**Conclusions:**

Our findings indicate a pronounced negative impact of IDD on QoL, consistent with observations reported in recent literature. Furthermore, in alignment with a limited number of prior studies, we identified a significant association between IDD and psychiatric disorders, suggesting that patients with IDD are more likely to present with comorbid mood and anxiety disorders.

**Disclosure of Interest:**

None Declared

